# The shape of telephone cord blisters

**DOI:** 10.1038/ncomms14138

**Published:** 2017-01-20

**Authors:** Yong Ni, Senjiang Yu, Hongyuan Jiang, Linghui He

**Affiliations:** 1 CAS Key Laboratory of Mechanical Behavior and Design of Materials, Department of Modern Mechanics, University of Science and Technology of China, Hefei, Anhui 230026, China; 2 Department of Physics, China Jiliang University, Hangzhou 310018, China

## Abstract

Formation of telephone cord blisters as a result of buckling delamination is widely observed in many compressed film-substrate systems. Here we report a universal morphological feature of such blisters characterized by their sequential sectional profiles exhibiting a butterfly shape using atomic force microscopy. Two kinds of buckle morphologies, light and heavy telephone cord blisters, are observed and differentiated by measurable geometrical parameters. Based on the Föppl-von Kármán plate theory, the observed three-dimensional features of the telephone cord blister are predicted by the proposed approximate analytical model and simulation. The latter further replicates growth and coalescence of the telephone cord into complex buckling delamination patterns observed in the experiment.

## Abstract (web summary)

Telephone cord blisters constitute a well-known example of patterns generated following buckling in thin films. Here the authors develop an analytical approach that can model the sectional height profiles along the blisters that they measure experimentally and simulate numerically.

Detailed origins of postbuckling phenomena have been a subject of discussions for several decades^1^. The buckles usually involve multiple instabilities and exhibit intriguing morphologies^2^,^3^. When a film deposited on a substrate is subject to a large residual compression, it buckles and delaminates away from the substrate, and the blisters with straight-sided, circular, telephone cord (TC) or network-like patterns are formed^4^,^5^. Predicting the shape of such blisters is of scientific interest for stress-driven pattern formation and important for avoiding structural failure in various film-substrate systems^4^. Among various manifestations of this phenomenon, the TC blister is the most frequently observed in many different film-substrate systems^5^. This motivates a search to identify universal features of TC blisters. Recently, TC formation has been considered as a development from a secondary instability of straight-sided or circular blisters. The instability condition has been analytically addressed by the Föppl-von Kármán (FvK) plate model^6^,^7^,^8^,^9^. Further, it has been found that the TC blister can also grow with the appearance of sags by an oscillatory process and its edge unnecessarily starts straight^10^,^11^. So far, a convincing solution for the three-dimensional (3D) TC blister morphology remains a challenge because of limited methods for handling nonlinear elasticity of thin plate, although a series of numerical models and experiments have provided valuable insights into the morphological features of TC blisters^10^,^11^,^12^,^13^,^14^,^15^,^16^,^17^,^18^,^19^,^20^,^21^,^22^,^23^,^24^,^25^.

The elegant instability analysis of a straight-sided blister predicted a constant undulation-period-to-width ratio of the TC buckle under the clamping boundary conditions close to the experimental observation^8^. In addition, the ratio may be not constant and change with the interfacial adhesion and the film-to-substrate modulus ratio^11^,^12^,^13^,^14^,^15^,^16^. The zigzag undulation feature of the TC buckle has been replicated by numerical investigations^5^,^15^,^16^,^17^,^18^,^19^. A comprehensive study for the 3D TC morphology suggested that it could be approximately viewed as a sequence of connected segments of a circular buckle pinned at its centre^12^. However, such approximation predicted the ridge of the TC blister to be discontinuous, different from our following refined experimental observations wherein the ridge is continuous and its height is periodically changing.

Here we aim to probe the 3D features of the TC morphology using a combination of analytical model, numerical simulation and atomic force microscopy (AFM) study. As shown in Fig. 1a,b, two typical TC blister shapes, light and heavy TC blisters, can be observed in compressed SiAlN_*x*_ films on glass substrates. Our experimental observation based on the optical images in Fig. 1a,b indicates that the projected area of the delamination zone of the TC blister is similar to the area swept by a segment of width 2*b* perpendicularly to a sinusoidal centreline. We then assume that the shape of the TC blister can thus be modelled as the postbuckling morphology of the thin plate clamped along such delamination boundary under equal biaxial residual compression (Fig. 1c). An approximate analytical solution for the shape of the TC blister is obtained in the following part. We argue that the shape of the TC blister can be characterized by several sectional profiles perpendicular to the centreline of the delamination zone (Fig. 1c). In contrast to the fact that the sectional profile of the straight-sided blister is always symmetric, the corresponding profile of the TC blister becomes asymmetric, dependent on the wavy amplitude of the centreline. The sequential sectional profiles of the TC blister exhibit a butterfly shape. Such geometric feature becomes more significant in the case of larger waviness amplitude of the centreline, corresponding to a transition from the light TC blister to the heavy TC blister.

## Results

### Analytical solution to the 3D profile of TC blisters

**Figure 1:**
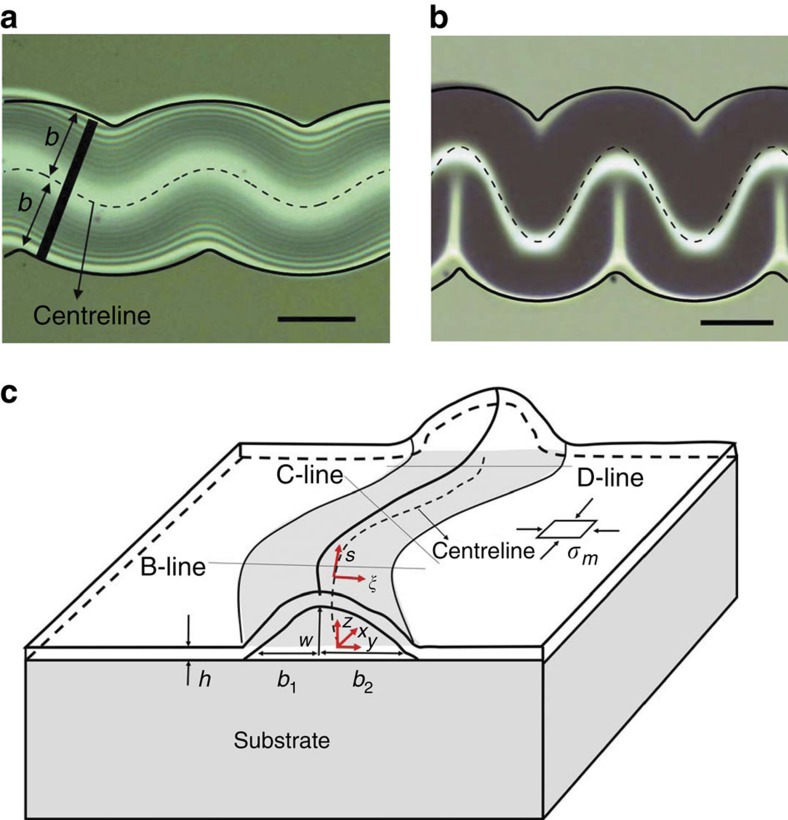
Schematic model for the shape of telephone cord blisters. Fitting the projected area of (**a**) a light TC blister and (**b**) a heavy TC blister observed in SiAlN_*x*_ films on glass substrates. (**c**) Sketch of the FvK model in a curvilinear coordinate under the clamping boundary condition along the boundary of the projected area. Scale bars in (**a**) and (**b**) are 30 and 50 μm, respectively.

We firstly determine the 3D shape of the TC blister by solving the FvK equations for a plate of thickness *h* clamped along the delamination boundary in a curvilinear coordinate (Fig. 1c). The centreline of the delamination zone is described by 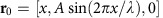, where *A* and *λ* are the amplitude and the wavelength of the wavy centreline, respectively. The shape of the TC buckles reflected by the profile of the mid-plane of the thin plate after deformation can be expressed as


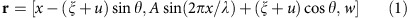


where *θ* (*x*) is defined as the angle between the tangential of the centreline and *x* axis, (*s*, *ξ*) is a curvilinear coordinate, *u*(*s*, *ξ*) is the displacement along *ξ* direction and *w*(*s*, *ξ*) is the deflection of the plate. Here we have assumed that the displacement change along the *S* direction is negligible compared to *u*(*s*, *ξ*) and *w*(*s*, *ξ*). The edges of the TC buckles are clamped at *ξ*=±*b*. We also assume that the in-plane displacement is small, that is, 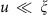, 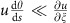, and the out-of-plane deflection is relatively large. Based on the observed morphology of the TC buckles, we make another important assumption that *u* and *w* change slowly in the *S* direction, that is, 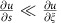 and 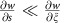. We derive the FvK equations with the boundary condition in the curvilinear coordinate by minimizing the total elastic energy in the plate (see the details in the Supplementary Note 1 for the analytical solution to the TC buckle). The strain energy of the TC buckle within a period can be written as


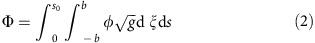


where *g* is the determinant of the metric tensor *g*_*αβ*_ and therefore 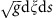 is the element of the area, *φ* is the elastic strain energy density per unit area having the form


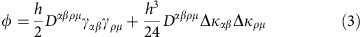


where *γ*_*αβ*_, Δ*k*_*αβ*_ and 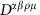 are defined as the mid-plane Lagrange strain tensor, the curvature change tensor due to deformation and the tensor of the elastic constants in the curvilinear coordinate, respectively. They can be explicitly expressed as a function of the variables *s*, *ξ*, *u* and *w*. Here the prime denotes differentiation with respect to *ξ*. If we only keep the leading order term of 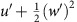, the element of area 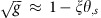 with 
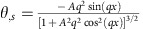
. The arc length of the centreline *s*(*x*) is given by 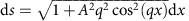, with *q*=2*π*/*λ*. We also assume that *s*=0 at *x*=0 and *s*=*s*_0_ at *x*=2*π*/*q*. From 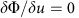, we have the in-plane equilibrium equation of the plate


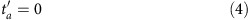


where 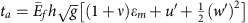 is the membrane stress along the direction of *ξ* in the film. 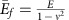 is plane strain modulus with *E*, *v* the Young's modulus and the Poisson's ratio of the plate, respectively. *ɛ*_*m*_ is the uniform equibiaxial residual strain in the plate. Equation (4) indicates the membrane stress *t*_*a*_ should be a constant. Similarly, from 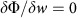 we obtain the out-of-plane equilibrium equation of the plate


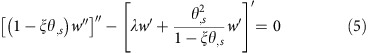


where 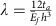. By solving equations (4) and (5) as an eigenvalue problem, we get an approximate solution to the deflection of the plate up to the first order


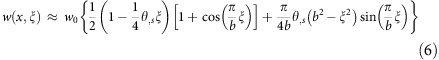


where *w*_0_ is an unknown constant. Equation (6) is reduced to 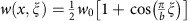 for the buckling of a straight strip with uniform width (*θ*_,*s*_=0) (ref. 6). Therefore, *w*_0_ represents the maximum deflection of the plate when *θ*_,*s*_=0. Note that 
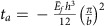
 at *θ*_,*s*_=0. Integrate this equation from −*b* to *b*, and notice that *u*=0 at *ξ*=±*b*, one can get


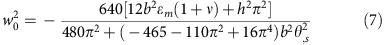


### The characteristic shape of TC blisters

**Figure 2:**
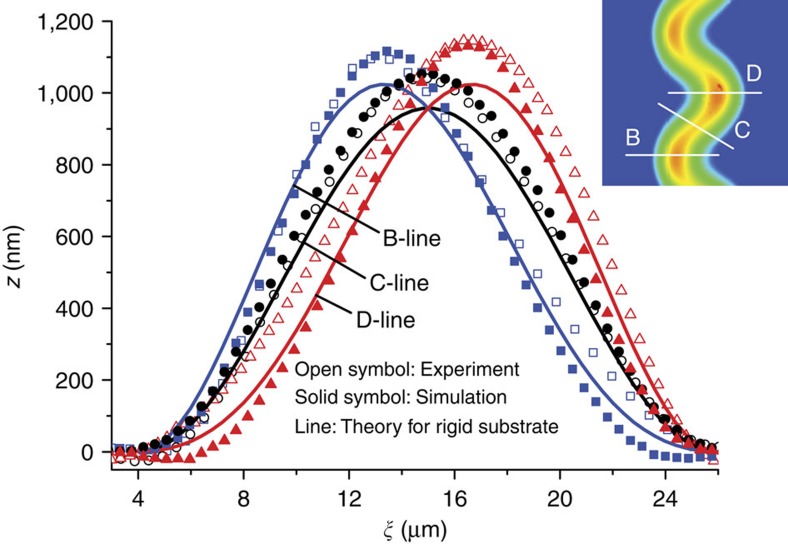
Characterization of the TC blister shape. Several sectional profiles perpendicular to the centreline are shown in the inserted modified AFM image 40 μm × 40 μm, which is updated by manually performing planefit offline of the original AFM image obtained by line-by-line (256 lines) scanning modes. The results from theory, simulation and AFM measurements are compared in a Ta film on glass substrates.

Equation (6) together with equation (7) provides for the first time an approximate analytical solution to the 3D profile of the TC buckle after the specific delamination area is available. To test whether the solution can predict the 3D feature of the TC blister shape or not, Fig. 2 plots several sectional profiles of a buckled tantalum (Ta) film on glass substrates perpendicular to the centreline shown in the inserted modified AFM image with the size of 40 μm × 40 μm. The result in Fig. 2 shows that the sequence of the sectional profiles of the TC blister perpendicular to the centreline exhibits a butterfly shape reminiscent of the butterfly curve of strain versus applied load in shape memory alloys. The asymmetry is characterized by two different half-separations denoted by *b*_1_ and *b*_2_ based on the ridge line of the TC blister (see Fig. 1c). The asymmetry has a maximum in the profile along B-line or D-line, while the profile along the C-line is symmetric. In addition, the maximum deflection in each sectional profile corresponding to the point at the ridge line is not constant. It has the smallest value in the profile along the C-line and the largest value in the profile along the B-line or D-line. This geometric feature of the TC blister is different from the previous report^12^ wherein the ridge line is viewed as a discontinuous contour line. Figure 2 demonstrates that the theoretical result obtained by equation (6) predicts the asymmetric 3D feature of the TC blister, and the deflection amplitude variation of the TC ridge line. This is consistent with the AFM measurements after the measured parameters of the buckled Ta film is given as *h*=225 nm, *b*=10 μm, *λ*/*b*=2, *A*/*λ*=0.08, and the residual strain in the Ta film is assumed to be *ɛ*_*m*_=0.004. The deflection amplitude of the ridge line given by equation (6) is underestimated because of the assumption of rigid substrate and clamping boundary condition. In fact, the substrate compliance could increase the maximum deflection^26^. We further numerically track the postbuckling morphology of the compressed plate on the substrate by setting the same predelamination zone, the same residual stress and the same other material parameters as those in the case of the analytical solution. The film-to-substrate modulus ratio is set to be 2.5 (about 70 GPa for the glass substrate and 175 GPa for Ta film) based on the continuum model^15^. The numerical result in Fig. 2 matches the experimentally observed morphological feature of the TC blister even more closely.

**Figure 3:**
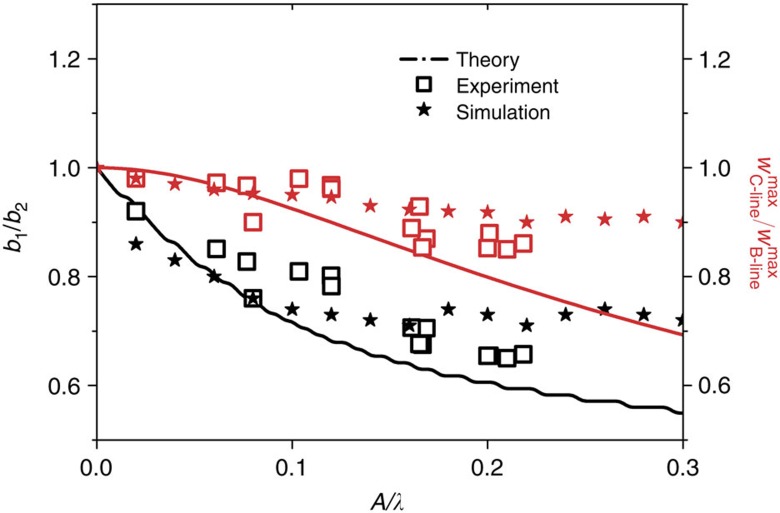
Measurable geometrical parameters. *b*_1_/*b*_2_ and 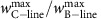 as a function of *A*/*λ* characterize different undulatory shapes of the TC blisters: comparison between theory, simulation and experimental observations in various films including Ta, SiAlN_*x*_, Fe and Ni on glass substrates.

The approximate solution in equation (6) also predicts the asymmetry denoted by *b*_1_/*b*_2_ and the variation of the maximum deflection at the ridge of the TC blister denoted by 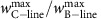 as a function of the waviness of the centreline denoted by *A*/*λ*. In the limit of *A*/*λ*=0, *b*_1_/*b*_2_=1 and 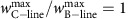, it is the case of a straight-sided blister. The stability analysis for the straight-sided blister only predicted the onset stage during formation of the TC blister, namely, the amplitude of the undulation is infinitely small corresponding to *A*/*λ*→0 (ref. 8). The current solution can apply to the case with finite value of *A*/*λ*. Figure 3 shows that different undulatory shapes of the TC blisters can be described by two measurable geometrical parameters *b*_1_/*b*_2_ and 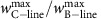 as a function of *A*/*λ* with the comparison between theory, simulation and experimental observations in various films including Ta, SiAlN_*x*_, Fe and Ni on glass substrates. With the increase of *A*/*λ*, the values of *b*_1_/*b*_2_ and 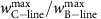 more significantly deviate from one, corresponding to the larger asymmetry. However, we must note that the approximate analytical solution is reliable only when the value of *A*/*λ* is very small since it proceeds by an asymptotic expansion in *θ*_,*s*_ (see the details in the Supplementary Note 1 for analytical solution to the TC buckle). From equation (5) it indicates that the solution may be questionable since there is a singularity as 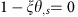. The singularity disappears when 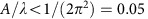 at *λ*=2*b*. For the larger value of *A*/*λ*, the numerical solution^15^ is more close to the experimental data, as shown in Fig. 3. It is found that the variations of *b*_1_/*b*_2_ and 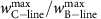 tend to be saturated, respectively, after *A*/*λ*>0.08, in contrast to the monotonous deviation from one predicted by the approximate analytical solution. The result in Fig. 3 thus demonstrates that *b*_1_/*b*_2_ and 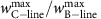 are another two measurable geometric parameters to characterize the undulatory morphology of the TC blister beside of *λ*/*b* and *A*/*λ*. The dependence of *b*_1_/*b*_2_ and 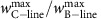 on *A*/*λ* is attributed to the fact that the wavy centreline of the delamination zone breaks the symmetry with respect to the straight centreline and introduces a position-dependent curvature as indicated in Supplementary Equation (10) in the buckled film wherein the buckling across the centreline may be not symmetric anymore.

### Light and heavy TC blisters

**Figure 4:**
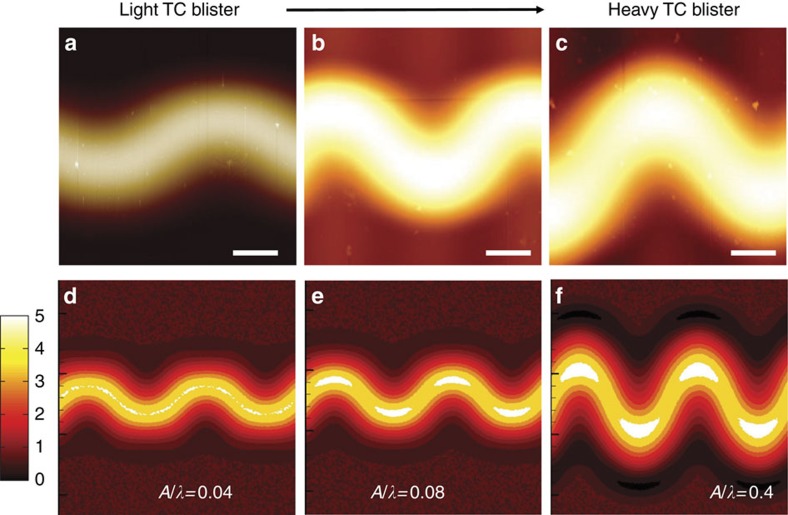
Morphologies of light and heavy TC blisters. (**a**–**c**) AFM images. Scale bars are 10 μm. (**d**–**f**) Numerical simulation results with parameters *b*/*h*=49, *λ*/*b*=2, *ɛ*_*m*_=0.005, and *A*/*λ*=0.04, 0.08 and 0.4.

Figure 4 demonstrates that there are two kinds of buckle morphologies, light and heavy TC blisters, differentiated by the value of *A*/*λ*. The upper row in Fig. 4 is the experimental observation in Ta and SiAlN_*x*_ films on glass substrates, respectively. The lower row in Fig. 4 is the simulated postbuckling morphology given *b/h*=49, *λ*/*b*=2 and 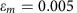 at different values of *A*/*λ*. It is found that with the increase of *A*/*λ*, the buckle really turns out to be ubiquitously undulated from light to heavy TC blisters. The obtained result is consistent with the experimental observation shown in Supplementary Movie 1, where an initially straight blister progressively grows into the familiar TC structure. We believe that the transition point between the light and heavy TC blisters depends on the value of *A*/*λ*. In the light TC blister, the value of *A*/*λ* is very small, the width of the delamination area is close to 2*b*, the delamination boundary is smooth and the values of *b*_1_/*b*_2_ and 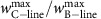 monotonously deviate from one. In contrast, in the heavy TC blister, the value of *A*/*λ* is much larger, the delamination zone significantly widens, the values of *b*_1_/*b*_2_ and 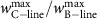 are saturated, and the delamination boundary shows a cusp indicating the existence of a singularity (see Supplementary Fig. 3).

In our analytical approach, *λ*/*b* and *A*/*λ* extracted from the projected area of the TC blister are assumed to be the input parameters instead of the prediction. Based on the assumption, the equilibrium values of *b*_1_/*b*_2_ and 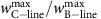 that quantify the typical 3D features of the TC blister are determined by the postbuckling solution given delamination area. We can obtain the values of *λ*/*b* and *A*/*λ* from the experimental observation. The measurement of *λ*/*b* is straightforward^12^. While the measurement of *A*/*λ* could be obtained by tracking the undulation of the ridge line denoted by 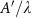, which value could be roughly viewed as that of *A*/*λ* (Fig. 1a,b). The results shown in Supplementary Fig. 2 indicate that both values of *λ*/*b* and 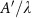 in various observed TC blisters tend to be saturated and fall into a narrow range. The value of the former is between 1.6 and 2.8, consistent with the report in the literature^16^. The value of the latter is between 0.1 and 0.3. Since the TC blister can be developed from a secondary buckling instability of a straight-sided blister, where the instability mode predicts *λ*/*b*≈2 given the parameters *b* and *A*=0 (ref. 8), we believe that the waviness period of the TC blister may be inherited from the instability wavelength. The deviation from *λ*/*b*≈2 is attributed that *λ*/*b* also depends on the adhesion with a non-trivial relationship^14^,^16^. Usually the increase of *A*/*λ* releases more the elastic energy in the biaxially compressed film deposited on the substrate, while it has to increase the mixed-mode-dependent adhesion energy. The competition between them sets the equilibrium value of *A*/*λ*. In addition, large value of *A*/*λ* in the TC blisters is hard to report. It is possible because the case with a large value of *A*/*λ* definitely increases the stress concentration which may lead to ridge crack^20^ or further buckling bifurcation, and the buckle is not TC blister any more^15^.

Up to now, our modelling to the shape of the TC blister is obtained under the particular assumption on the shape of the delamination region. In fact, the delamination area is unnecessary to be sinusoidal shaped, especially during the oscillatory growth process of the TC blister^11^. Usually, determination of the buckle–delamination morphology can be separated into two steps. The first step is to derive a postbuckling solution given the delamination zone. The second step is to determine the delamination zone at equilibrium. If the edge of the delamination zone is described as the interfacial crack front, the delamination zone at equilibrium is determined as the buckling-mediated energy release rate is equal to the interface toughness. For the straight-sided blister, both steps are done analytically^6^. However, the analytical solution to the second step for the TC blister is not available.

In fact, both the equilibrium delamination area and postbuckling morphology in thin films deposited on substrates can also be theoretically determined in principle. If we further take into account the contribution of the adhesion energy and the elastic strain energy in the substrate, all the equilibrium values of *λ*/*b*, *A*/*λ*, *b*_1_/*b*_2_ and 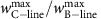 that quantify the TC blister shape with less restriction can be numerically determined by minimizing the total free energy in the film-substrate system. Our current numerical simulations rely on the recently developed continuum modelling to track the general morphological evolution of the buckle delamination without any restriction of the delamination zone^15^. In this approach, the concurrent buckling and delaminating processes are formulated using the time-dependent Ginzburg–Landau kinetic equations, driven by minimizing the film-substrate total free energy, including the elastic energies in both the film and the substrate, and the mixed-mode interfacial adhesion between them^11^,^15^. The effect of substrate elasticity and interfacial adhesion on the shape of the TC blister can be taken into account, which is neglected in the analytical approach. The modelling and simulation approach is outlined in Supplementary Note 3 and some results are shown in Supplementary Figs 4–6. The coupling behaviour between buckling and delamination in a film deposited on substrates with higher compressive stresses becomes more complex^20^,^22^, and the TC blister may exhibit beyond the sinusoidal configuration. Our numerical simulations not only recover the growth process of the TC blister from an initially circular blister^7^ but also capture a rich coalescence behaviour accompanied with the increase of the buckling width during further propagation of the TC buckle. The TC buckle becomes larger and larger with the appearance of several spikes and/or daughter TC buckle at the outer undulated edge, consistent with our experimental observation (see Supplementary Fig. 6 and Supplementary Movies 2 and 3).

## Discussion

In summary, the refined 3D morphological features of the TC buckle are elucidated by using AFM characterization, approximate analytical model and numerical simulations. We confirm that the shape of the TC blister can be modelled as the postbuckling morphology of the compressed plate clamped along the delamination front given the parameters *λ*/*b* and *A*/*λ*. Two measurable geometrical parameters *b*_1_/*b*_2_ and 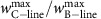 are proposed to characterize a so-called ‘butterfly shape' of the sequential sectional profiles, which are universal in both the light and heavy TC blisters. The above features are successfully reproduced by our approximate analytical model and numerical simulations. Furthermore, how the fully nonlinear buckle-driven delamination process leads to the morphological evolution of the TC blister is captured by numerical simulations and experimental observation. The present work provides insight into the 3D shape of TC buckles.

## Methods

Formation of the TC buckle as a result of the buckle-driven delamination process was simulated using the phase field method where the film buckles into an equilibrium buckle–delamination configuration driven by minimizing the total free energy. The total free energy of the film-substrate system is established by incorporating Green function method for the substrate elasticity, FvK plate theory for nonlinear film deformation and cohesive zone model for mixed-mode interfacial adhesion. The total free energy of the film-substrate system including the film, the substrate and the interface can be expressed as a functional of the out-of-plane displacement of the film *ζ*(**x**, *t*) and the displacement jump vector across the interface 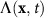 as 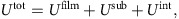 where *U*^film^ is the elastic strain energy including the bending and stretching energies in the film, *U*^sub^ is the elastic energy of the substrate and *U*^int^ is the adhesion energy between the film and the substrate. Their detailed expressions are found in ref. 15. Following ref. 11, we may also adopt alternative cohesive zone model with bilinear traction versus separation law for better description of mixed-mode dependence of interface adhesion. The dynamic equations for 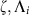 denoted as the time-dependent Ginzburg–Landau kinetic equations to describe the minimization process of the total free energy are given by: 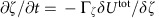 and 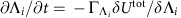, where *t* denotes time and 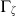, 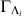
are the kinetic coefficients that characterize the relaxation rates of the buckling and delamination processes in the overdamped dynamics. This gradient flow form of the dynamics guarantees a monotonous minimization process of *U*^tot^ and ensures a convergent solution of the dynamic equations, whose steady-state solutions provide the configuration at the equilibrium. We solve the reduced forms in a computational cell with periodic boundary conditions using input parameters: 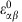, 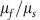, 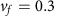, 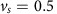, 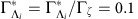, 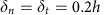, 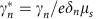 and 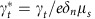. The grid spacing is 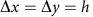 and the time step is 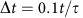, with 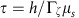. A small random fluctuation mimicking the thermal fluctuation is used to facilitate nucleation of buckling delamination from the pre-existing interfacial delamination region, in which zero interface toughness is assumed.

### Data availability

The data that support the findings of this study are available from the corresponding author on request.

## Additional information

**How to cite this article:** Ni, Y. *et al*. The shape of telephone cord blisters. *Nat. Commun.* **8,** 14138 doi: 10.1038/ncomms14138 (2017).

**Publisher's note:** Springer Nature remains neutral with regard to jurisdictional claims in published maps and institutional affiliations.

## Supplementary Material

Supplementary Information Supplementary Figures, Supplementary Notes and Supplementary References

Supplementary Movie 1 In situ evolution of the telephone cord buckle in a SiAlNx film from a straight-sided blister. The formation and propagation of the telephone cord buckle are displayed by an optical microscope (Leica DMLM) equipped with a charge coupled device camera (Leica DC 300). The SiAlNx film deposited on rectangular float glass substrates was made by using an off-line coating production line (Apollon G 3210/7-H, Leybold). The film thickness is about 400 nm. After annealing, the release of high compressive stress in the film drives nucleation of a straight-sided blister at some delamination defects. After the straight-sided blister loses its stability, it progressively changes from a light telephone cord buckle towards a heavy telephone cord buckle.

Supplementary Movie 2 Simulated evolution of the telephone cord buckle. The simulated evolution of an initial straight-sided blister into complex buckle-delamination patterns validated by the experimental observation in Movie 3. After the secondary buckling instability occurs, the straight-sided blister tends to be the telephone cord buckle. Further propagation of the buckle shows a rich coalescence behavior accompanied with the increase of the buckling width. The telephone cord buckle becomes larger and larger with the appearance of several spikes and/or daughter telephone cord buckle at the outer undulated edge. The simulated result is obtained by numerically solving the above outlined continuum model in a computational cell of size 256h×256h with periodic boundary conditions using input parameters *ε*^0^_αβ_=*ε*_*m*_ δ_αβ_, *ε*_*m*_=0.03 μ_*f*_/μ_*s*_=4, *v*_*f*_=0.3, *v*_*s*_=0.5,Γ^*^_Λ_*i*__=Γ_Λ_*i*__/Γ_ζ_=0.01, δ_*n*_=0.2*h*, δ_*t*_=2δ_*n*_, γ^*^_n_=γ_*n*_/*e*δ_*n*_μ_*s*_=0.019 and γ^*^_t_=γ_*t*_/*e*δ_*n*_μ_*s*_=0.019.

Supplementary Movie 3 In situ evolution of the telephone cord buckle with coalescence behavior. The growth of the telephone cord buckle with interesting coalescence behavior consistent with the simulated result in Movie 2 is observed in the annealed SiAlNx film deposited on float glass by an optical microscope (Leica DMLM) equipped with a charge coupled device camera (Leica DC 300). The film is obtained by using the same method as that in Movie 1 but with different annealing time.
