# Supplementary material for: The shape of telephone cord blisters
Source: Nat Commun. 2017 Jan 20;8:14138. doi: 10.1038/ncomms14138 (PMC5263886; doi:10.1038/ncomms14138)
Supplement: Supplementary Information — Supplementary Figures, Supplementary Notes and Supplementary References [file ncomms14138-s1.pdf]

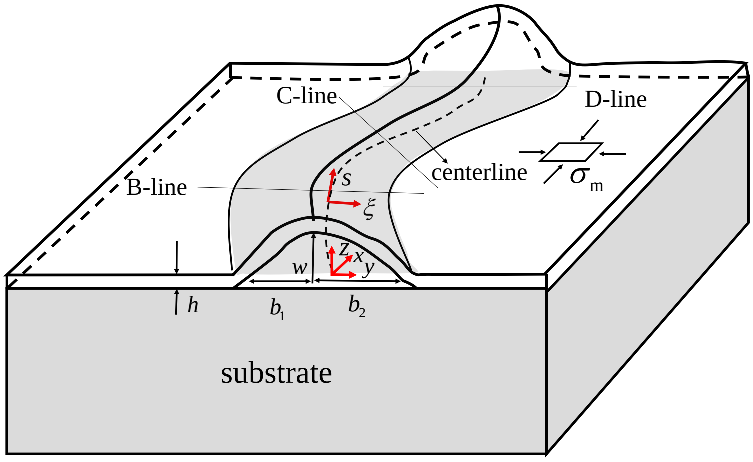

Supplementary Figure 1. sketch of the FvK model in a curvilinear coordinate under the clamping boundary condition along the boundary of the projected area.

(a)

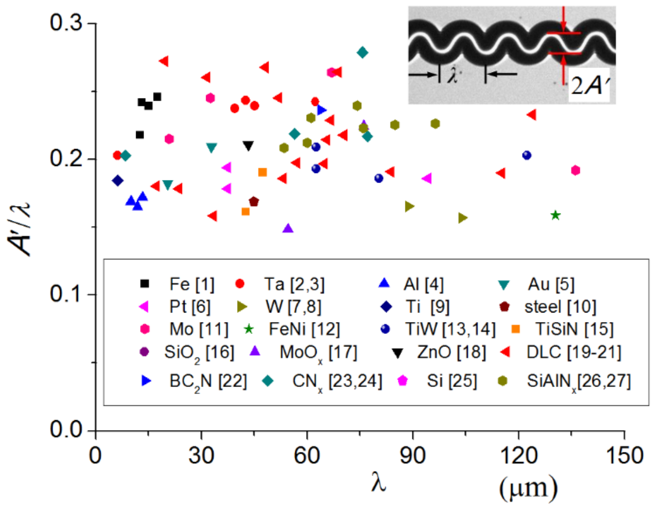

(b)

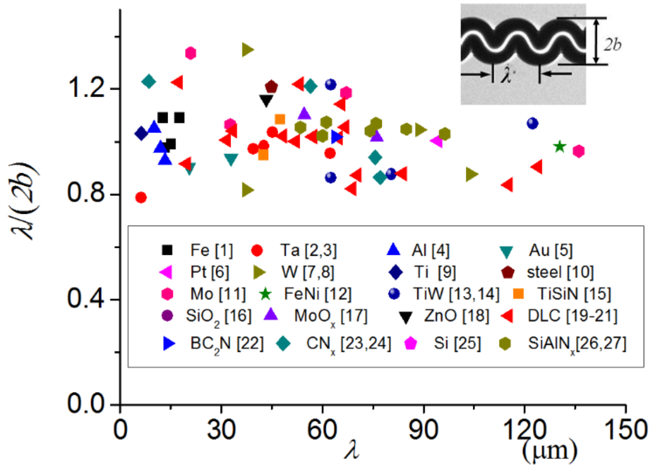

Supplementary Figure 2. Plot of A'/λ and $\lambda/(2b)$ as a function of λ in various TC blister buckles

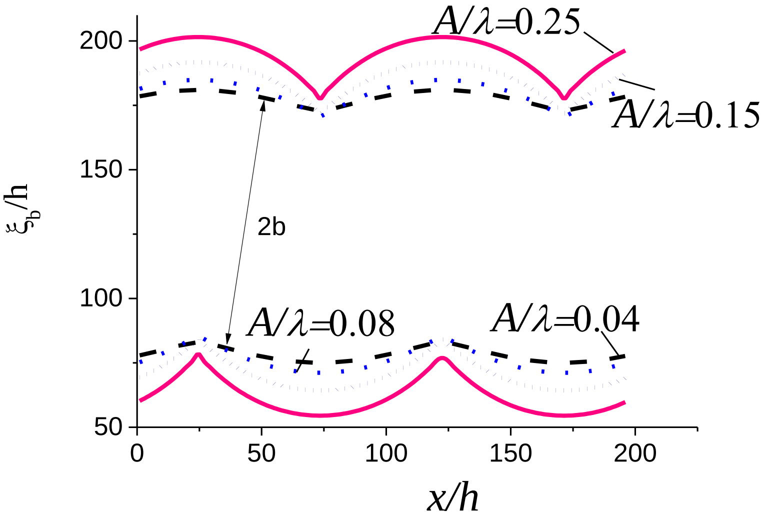

Supplementary Figure 3. Plot of the side undulation curve under different values of A/λ

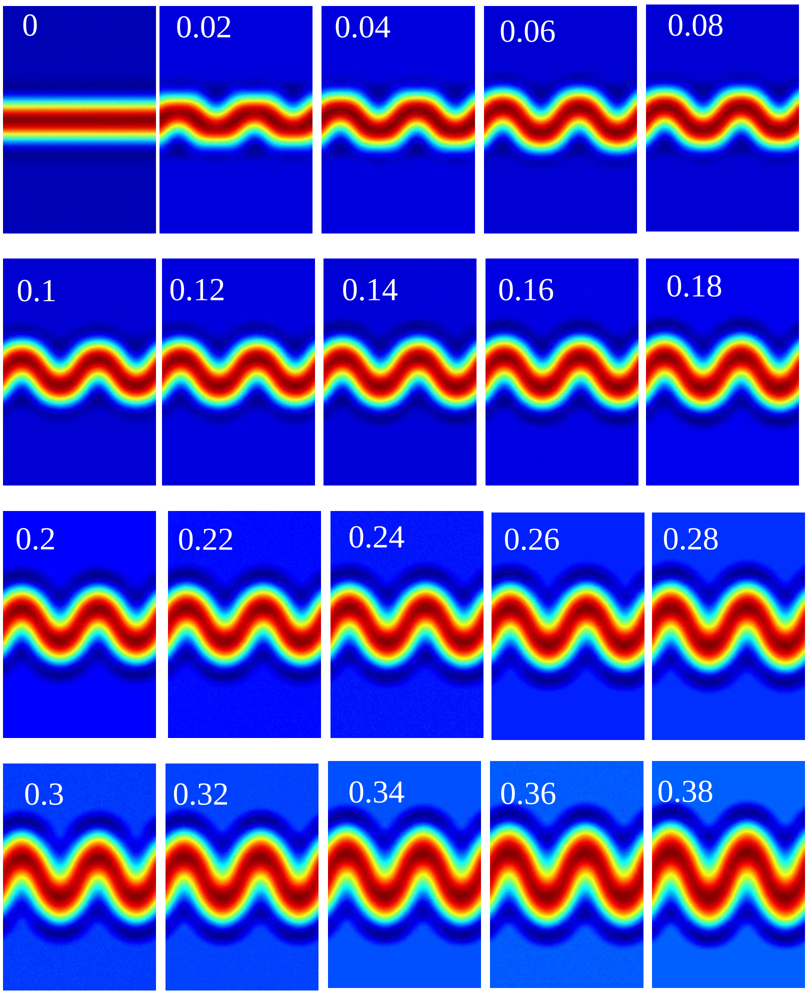

Supplementary Figure 4. The simulated contour map of the post-buckling morphology under different value of A/λ from 0 to 0.38 with the increase of 0.02

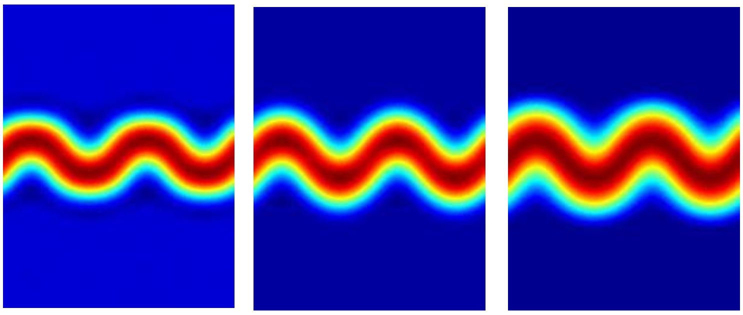

$\frac{\mu_f}{\mu_s} = 2$ $\frac{\mu_f}{\mu_s} = 5$ $\frac{\mu_f}{\mu_s} = 20$

Supplementary Figure 5. Effect of the modulus ratio between film and substrate on the shape of TC blister

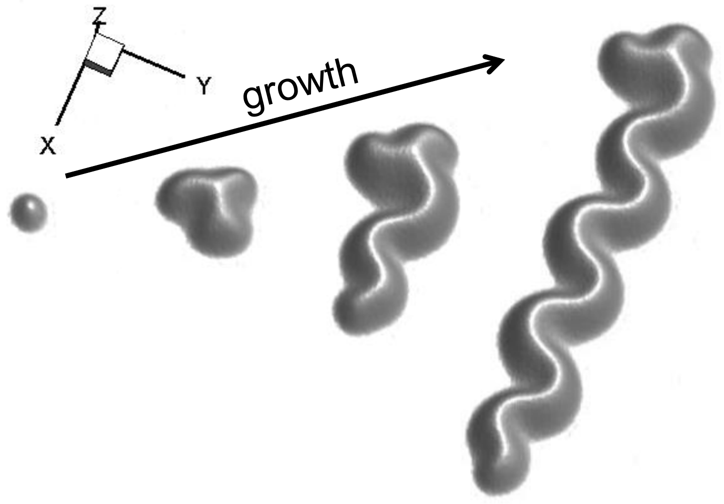

Supplementary Figure 6. The growth of a TC blister obtained by numerical simulation

Supplementary Note 1: Analytical solution to the telephone cord buckle

As shown Supplementary Figure 1, the centerline of the telephone cord can be given by $\mathbf{r}_0 = \left[x, A\sin\left(2\pi x/\lambda\right), 0\right]$ where A and λ are the amplitude and wavelength of the centerline, respectively. The tangent vector of the centerline is $\frac{d\mathbf{r}_0}{dx} = \left[1, -Aq\cos\left(qx\right), 0\right]$ with $q = 2\pi/\lambda$. Therefore, the distance between the two nearby points is

$$ds^2 = \frac{d\mathbf{r}_0}{dx}\cdot\frac{d\mathbf{r}_0}{dx} = \left[1 + A^2q^2\cos^2\left(qx\right)\right]dx^2 \tag{S.1}$$

Define the angle $\theta(x)$ between the tangential of the centerline and x axis so that

$$\begin{aligned}\cos\theta &= \frac{dx}{ds} = \frac{1}{\sqrt{1 + A^2q^2\cos^2\left(qx\right)}}\\ \sin\theta &= \frac{dy}{ds} = \frac{Aq\cos\left(qx\right)}{\sqrt{1 + A^2q^2\cos^2\left(qx\right)}}\end{aligned} \tag{S.2}$$

Therefore, the shape of the telephone cord buckles can be described as

$$\mathbf{r} = \left[x - \left(\xi + u\right)\sin\theta, A\sin\left(qx\right) + \left(\xi + u\right)\cos\theta, w\right] \tag{S.3}$$

where $\left(s, \xi\right)$ is a curvilinear coordinate and $u\left(s, \xi\right)$ is the displacement along ξ direction and $w\left(s, \xi\right)$ is the deflection of the plate. Here, we have assumed that the displacement along the s direction is negligible compared to $u\left(s, \xi\right)$ and $w\left(s, \xi\right)$. The edge of the telephone cord buckles are clamped at $\xi = \pm b$. By considering Eq. (S.2), the local tangential vectors can be written as

$$\begin{aligned}\mathbf{m}_1 &= \frac{\partial \mathbf{r}}{\partial s} = \left[\left[1 - \left(\xi + u\right)\frac{d\theta}{ds}\right]\cos\theta - \frac{\partial u}{\partial s}\sin\theta, \left[1 - \left(\xi + u\right)\frac{d\theta}{ds}\right]\sin\theta + \frac{\partial u}{\partial s}\cos\theta, \frac{\partial w}{\partial s}\right]\\ \mathbf{m}_2 &= \frac{\partial \mathbf{r}}{\partial \xi} = \left[-\left(1 + \frac{\partial u}{\partial \xi}\right)\sin\theta, \left(1 + \frac{\partial u}{\partial \xi}\right)\cos\theta, \frac{\partial w}{\partial \xi}\right]\end{aligned} \tag{S.4}$$

In this work, we assume the in-plane displacement is small, i.e., $u \ll \xi$ and

$u\frac{d\theta}{ds} \ll \frac{\partial u}{\partial \xi}$, and the out-of-plane deflection is relatively large. From the morphology of telephone cord buckles, we make another important assumption that u and w change slowly in the s direction, i.e., $\frac{\partial u}{\partial s} \ll \frac{\partial u}{\partial \xi}$ and $\frac{\partial w}{\partial s} \ll \frac{\partial w}{\partial \xi}$. Therefore, the two tangential vectors can be simplified to

$$\begin{aligned} \mathbf{m}_1 &= \left[\left(1-\xi\theta_{,s}\right)\cos\theta, \left(1-\xi\theta_{,s}\right)\sin\theta, 0\right] \\ \mathbf{m}_2 &= \left[-\left(1+u'\right)\sin\theta, \left(1+u'\right)\cos\theta, w'\right] \end{aligned} \tag{S.5}$$

where $\theta_{,s} \equiv \frac{d\theta}{ds}$, $u' \equiv \frac{\partial u}{\partial \xi}$ and $w' \equiv \frac{\partial w}{\partial \xi}$. Here the prime denotes differentiation with respect to ξ. The metric tensor in the deformed film is given by

$$g_{\alpha\beta} = \mathbf{m}_\alpha \cdot \mathbf{m}_\beta = \begin{bmatrix} \left(1-\xi\theta_{,s}\right)^2 & 0 \\ 0 & \left(1+u'\right)^2 + \left(w'\right)^2 \end{bmatrix} \tag{S.6}$$

Similarly, we can calculate the tangential vectors and therefore obtain the metric tensor in the undeformed film as

$$\bar{g}_{\alpha\beta} = \begin{bmatrix} \left(1-\xi\theta_{,s}\right)^2 & 0 \\ 0 & 1 \end{bmatrix} \tag{S.7}$$

The mid-plane Lagrange strain tensor defined as

$$\gamma_{\alpha\beta} = \frac{1}{2}\left(g_{\alpha\beta} - \bar{g}_{\alpha\beta}\right) + \varepsilon_m \bar{g}_{\alpha\beta} = \begin{bmatrix} \varepsilon_m\left(1-\xi\theta_{,s}\right)^2 & 0 \\ 0 & \varepsilon_m + u' + \frac{1}{2}\left(w'\right)^2 \end{bmatrix} \tag{S.8}$$

where ε_m is the uniform equi-biaxial mismatch stress. Here we have used the assumption that $u' \ll 1$. The corresponding mixed component of the mid-plane Lagrange strain tensor is

$$\gamma_\alpha^\beta = \gamma_{\alpha\rho}\bar{g}^{\rho\beta} = \begin{bmatrix} \varepsilon_m & 0 \\ 0 & \varepsilon_m + u' + \frac{1}{2}\left(w'\right)^2 \end{bmatrix} \tag{S.9}$$

where $\bar{g}^{\rho\beta}$ is the inverse of the metric tensor in the undeformed film, i.e., $\bar{g}^{\alpha\rho}\bar{g}_{\rho\beta} = \delta^{\alpha}_{\beta}$. The curvature tensors in the deformed and undeformed film are

$$\kappa_{\alpha\beta} = \begin{bmatrix} -\theta_{,s} w'\left(1-\xi\theta_{,s}\right) & 0 \\ 0 & w'' \end{bmatrix} \quad \text{and} \quad \bar{\kappa}_{\alpha\beta} = 0 \tag{S.10}$$

Therefore, the curvature change tensor is

$$\Delta\kappa_{\alpha\beta} = \kappa_{\alpha\beta} - \bar{\kappa}_{\alpha\beta} = \begin{bmatrix} -\theta_{,s} w'\left(1-\xi\theta_{,s}\right) & 0 \\ 0 & w'' \end{bmatrix} \tag{S.11}$$

So the Lagrange strain tensor is defined as

$$E_{\alpha\beta} = \gamma_{\alpha\beta} + x_3 \Delta\kappa_{\alpha\beta} \tag{S.12}$$

with two nonzero components as

$$\begin{aligned} E_{11} &= \varepsilon_m \left(1-\xi\theta_{,s}\right)^2 - x_3\theta_{,s} w'\left(1-\xi\theta_{,s}\right) \\ E_{22} &= \varepsilon_m + u' + \frac{1}{2}\left(w'\right)^2 + x_3 w'' \end{aligned} \tag{S.13}$$

where x_3 is the distance from the mid-plane of the film. The tensor the elastic constants can be defined as

$$D^{\alpha\beta\rho\mu} = \frac{E}{2(1-\nu^2)}\left[\left(\bar{g}^{\alpha\rho}\bar{g}^{\beta\mu} + \bar{g}^{\alpha\mu}\bar{g}^{\beta\rho}\right)\left(1-\nu\right) + 2\upsilon\bar{g}^{\alpha\beta}\bar{g}^{\rho\mu}\right] \tag{S.14}$$

where E and ν are the Young's modulus and Poisson's ratio, respectively. The three nonzero components of $D^{\alpha\beta\rho\mu}$ are

$$\begin{aligned} D^{1111} &= \frac{E\left(\bar{g}^{11}\right)^2}{1-\nu^2} = \frac{E}{1-\nu^2}\frac{1}{\left(1-\xi\theta_{,s}\right)^4} \\ D^{2222} &= \frac{E\left(\bar{g}^{22}\right)^2}{1-\nu^2} = \frac{E}{1-\nu^2} \\ D^{1122} &= \frac{\nu E\bar{g}^{11}\bar{g}^{22}}{1-\nu^2} = \frac{\nu E}{1-\nu^2}\frac{1}{\left(1-\xi\theta_{,s}\right)^2} \end{aligned} \tag{S.15}$$

The stress-resultant tensor and the internal moment tensor are

$$
\begin{aligned}
T^{\alpha\beta} &= hD^{\alpha\beta\rho\mu}\gamma_{\rho\mu} \\
M^{\alpha\beta} &= \frac{h^3}{12}D^{\alpha\beta\rho\mu}\Delta\kappa_{\rho\mu}
\end{aligned}
\tag{S.16}
$$

Therefore, the elastic strain energy density per unit area is

$$
\begin{aligned}
\phi &= \frac{h}{2}D^{\alpha\beta\rho\mu}\gamma_{\alpha\beta}\gamma_{\rho\mu} + \frac{h^3}{24}D^{\alpha\beta\rho\mu}\Delta\kappa_{\alpha\beta}\Delta\kappa_{\rho\mu} \\
&= \frac{h}{2}\frac{E}{1-\nu^2}\left\{\varepsilon_m^2 + \left[\varepsilon_m + u' + \frac{1}{2}(w')^2\right]^2 + 2\nu\varepsilon_m\left[\varepsilon_m + u' + \frac{1}{2}(w')^2\right]\right\} \\
&\quad + \frac{h^3}{24}\frac{E}{1-\nu^2}\left[\frac{(\theta_{,s}w')^2}{(1-\xi\theta_{,s})^2} + (w'')^2 - \frac{2\nu\theta_{,s}w'w''}{1-\xi\theta_{,s}}\right]
\end{aligned}
\tag{S.17}
$$

The stain energy of the telephone cord buckles within a period is

$$
\Phi = \int_0^{s_0}\int_{-b}^{b}\phi\sqrt{g}\,d\xi ds \tag{S.18}
$$

where g is the determinant of the metric tensor $g_{\alpha\beta}$ and therefore $\sqrt{g}d\xi ds$ is the element of the area. If we only keep the leading order term of $u' + \frac{1}{2}(w')^2$, the element of area $\sqrt{g} \approx 1-\xi\theta_{,s}$. The arc-length of the centerline $s(x)$ is given by $ds = \sqrt{1+A^2q^2\cos^2(qx)}dx$. We also assume that $s=0$ at $x=0$ and $s=s_0$ at $x=2\pi/q$. From the principle of minimum potential energy, we can obtain the equilibrium equations. From $\delta\Phi/\delta u = 0$, we have

$$
t_a{}' = 0 \tag{S.19}
$$

where $t_a = \bar{E}_f h\sqrt{g}\left[(1+\nu)\varepsilon_m + u' + \frac{1}{2}(w')^2\right]$ is the in-plane normal force in the film. $\bar{E}_f = \frac{E}{1-\nu^2}$ is plane strain modulus. Eq. (S.19) indicates the in-plane normal force t_a should be a constant. Similarly, from $\delta\Phi/\delta w = 0$ we obtain another differential equation

$$
\left[(1-\xi\theta_{,s})w''\right]'' - \left[\lambda w' + \frac{\theta_{,s}{}^2}{1-\xi\theta_{,s}}w'\right]' = 0 \tag{S.20}
$$

where $\lambda = \frac{12 t_a}{\bar{E}_f h^3}$. Apparently, this is an eigenvalue problem. Here, we seek a perturbative solution to Eq. (S.20) of the form

$$\begin{aligned} \lambda_n &= \lambda_n^{(0)} + \theta_{,s} \lambda_n^{(1)} + \cdots \\ w_n &= w_n^{(0)} + \theta_{,s} w_n^{(1)} + \cdots \end{aligned} \tag{S.21}$$

where λ_n and w_n are the n-th eigenvalue and eigenfunction of Eq. (S.20). Substituting Eq. (S.21) into Eq. (S.20) and comparing the power of $\theta_{,s}$ gives the following sequence of equations:

$$\frac{\partial^4 w_n^{(0)}}{\partial \xi^4} - \lambda_n^{(0)} \frac{\partial^2 w_n^{(0)}}{\partial \xi^2} = 0 \tag{S.22}$$

$$\frac{\partial^4 w_n^{(1)}}{\partial \xi^4} - \lambda_n^{(0)} \frac{\partial^2 w_n^{(1)}}{\partial \xi^2} = \frac{\partial^2}{\partial \xi^2}\left(\xi \frac{\partial^2 w_n^{(0)}}{\partial \xi^2} \right) - \lambda_n^{(1)} \frac{\partial^2 w_n^{(0)}}{\partial \xi^2} \tag{S.23}$$

with boundary conditions

$$w_n^{(i)} = 0 \quad \text{and} \quad \frac{\partial w_n^{(i)}}{\partial \xi} = 0 \quad \text{at} \quad \xi = \pm b \tag{S.24}$$

Eq. (S.22) is the zeroth order approximation of the problem. In this case, the centerline is a straight line ($\theta_{,s} = 0$). The eigenvlaue and eigenfunciton of Eq. (S.22) are

$$\lambda_n^{(0)} = -\left(\frac{n\pi}{b}\right)^2 \tag{S.25}$$

$$w_n^{(0)} = \frac{1}{\sqrt{3b}}\left[(-1)^{n+1} + \cos\left(\frac{n\pi}{b}\xi\right) \right] \tag{S.26}$$

$w_n^{(0)}$ form an orthonormal basis, i.e.,

$$\int_{-b}^{b} w_m^{(0)} w_n^{(0)} d\xi = \begin{cases} 1 & (m = n) \\ 0 & (m \neq n) \end{cases} \tag{S.27}$$

By multiplying Eq. (S.23) by $w_m^{(0)}$ and integrating it from $-b$ to b, one can find

$$\lambda_n^{(1)} = -\frac{\int_{-b}^{b} w_n^{(0)} \frac{\partial^2}{\partial \xi^2}\left(\xi \frac{\partial^2 w_n^{(0)}}{\partial \xi^2}\right) d\xi}{\int_{-b}^{b} w_n^{(0)} \frac{\partial^2 w_n^{(0)}}{\partial \xi^2} d\xi} = 0 \tag{S.28}$$

when $m = n$. By substituting $\lambda_n^{(1)} = 0$ into Eq. (S.23) and solving the equation directly, one can get

$$w_n^{(1)} = \frac{1}{4\sqrt{3b}}\left\{\xi\left[(-1)^n - \cos\left(\frac{n\pi}{b}\xi\right)\right] + \frac{n\pi}{b}\left(b^2 - \xi^2\right)\sin\left(\frac{n\pi}{b}\xi\right)\right\} + C_0 w_n^{(0)} \tag{S.29}$$

where C_0 is an unknown constant. By multiplying Eq. (S.23) by $w_m^{(0)}$ ($m \neq n$) and integrating it from $-b$ to b, one can find

$$\left(\lambda_n^{(0)} - \lambda_m^{(0)}\right)\int_{-b}^{b} w_n^{(1)} \frac{\partial^2 w_m^{(0)}}{\partial \xi^2} d\xi = 0 \tag{S.30}$$

Notice that $\lambda_n^{(0)} \neq \lambda_m^{(0)}$ when $m \neq n$. Therefore, we get $\int_{-b}^{b} w_n^{(1)} \frac{\partial^2 w_m^{(0)}}{\partial \xi^2} d\xi = 0$, which indicates $C_0 = 0$. Therefore, when $n = 1$, we get an approximation of the deflection of the plate as

$$w(x,\xi) \approx w_0\left\{\frac{1}{2}\left(1 - \frac{1}{4}\theta_{,s}\xi\right)\left[1 + \cos\left(\frac{\pi}{b}\xi\right)\right] + \frac{\pi}{4b}\theta_{,s}\left(b^2 - \xi^2\right)\sin\left(\frac{\pi}{b}\xi\right)\right\} \tag{S.31}$$

where w_0 is an unknown constant. Eq. (S.31) is reduced to $w(x,\xi) \approx \frac{1}{2}w_0\left[1 + \cos\left(\frac{\pi}{b}\xi\right)\right]$ for the buckling of a straight strip with uniform width ($\theta_{,s} = 0$). Therefore, w_0 represents the maximum deflection of the plate when $\theta_{,s} = 0$.

Notice that $t_a = \bar{E}_f h\sqrt{g}\left[(1+\nu)\varepsilon_m + u' + \frac{1}{2}(w')^2\right] = \frac{\bar{E}_f h^3 \lambda_1}{12} = -\frac{\bar{E}_f h^3}{12}\left(\frac{\pi}{b}\right)^2$. Integrate this equation from $-b$ to b, and notice that $u = 0$ at $\xi = \pm b$, one can get

$$w_0^2 = -\frac{640\left[12b^2\varepsilon_m\left(1+\nu\right)+h^2\pi^2\right]}{480\pi^2+\left(-465-110\pi^2+16\pi^4\right)b^2\theta_{,s}^2} \tag{S.32}$$

Eq.(S.31) together with Eq.(S.32) provides an analytical solution to the 3D profile of the TC buckle after its projected area is available.

Supplementary Note 2: Measurable A'/λ and λ/b in various TC blisters shown in Supplementary Figure 2

We have measured the values of A'/λ and λ/b in various TC blisters according to the optical or AFM images reported in the literatures [1-27]. The values of A'/λ and λ/b for each kind of materials are estimated as the average by several measurements.

Supplementary Note 3: Method to simulate evolution of the telephone cord buckle

Supplementary Figure 3 shows the side undulation curve under different values of A/λ at b/h=49, $\lambda/b=2$ to set the pre-delamination area in our numerical simulation.

We perform the numerical simulation for the effect of A/λ on the post-buckling morphology of the compressed plate on the substrate given the parameters $\varepsilon_{\alpha\beta}^0=\varepsilon_m\delta_{\alpha\beta}$, $\varepsilon_m=0.005$ $\mu_f/\mu_s=2.5$, $\nu_f=0.3$, $\nu_s=0.5$, $\Gamma_{\Lambda_i}^*=\Gamma_{\Lambda_i}/\Gamma_\zeta=0.01$, b/h=49, $\lambda/b=2$, as shown in Supplementary Figure 4. The simulated result in Figure 3 about the dependence of b_1/b_2 and $w_{C-line}^{\max}/w_{B-line}^{\max}$ on A/λ is extracted from the data in Supplementary Figure 4.

Supplementary Figure 5 shows the effect of modulus ratio between film and

substrate on the post-buckling morphology of the compressed plate on the substrate given the parameters $\varepsilon_m = 0.005$, $v_f = 0.3$, $v_s = 0.5$, , b/h=49, $\lambda / b = 2$, A/λ=0.08. It is found that the larger modulus ratio leads to larger energy releasing rate and maximum deflection of the TC blister. Interestingly, the asymmetry characterized by $w_{C-line}^{\max} / w_{B-line}^{\max}$ becomes weak as the substrate is compliant, i.e. $\mu_f / \mu_s = 20$. This may indicate that it does not always happens. More detailed study is needed in future.

The simulated result in Supplementary Figure 6 is obtained by numerically solving the above outlined continuum model in a computational cell with periodic boundary conditions using input parameters: the cell size 2048h×2048h with an initial circular delamination nuclei of radius r=5h , $\varepsilon_{\alpha\beta}^0 = \varepsilon_m \delta_{\alpha\beta}$, $\varepsilon_m = 0.022$ $\mu_f / \mu_s = 5$, $v_f = 0.3$, $v_s = 0.5$, $\Gamma_{\Lambda_i}^* = \Gamma_{\Lambda_i} / \Gamma_\zeta = 0.01$, $\delta_n = 0.2h$, $\delta_t = \delta_n$, $\gamma_n^* = \gamma_n / e\delta_n\mu_s = 0.02$ and $\gamma_t^* = \gamma_t / e\delta_n\mu_s = 0.1$.

Supplementary References

[1] S. J. Yu, Y. J. Zhang, and M. G. Chen, Thin Solid Films **518**, 222 (2009).

[2] M. He, C. Gaire, G.C. Wang, and T.M. Lu, Micro. Reliab. **51**, 847 (2011).

[3] Q. L. Ye, and S. J. Yu, Philos. Mag. Lett. **93**, 710 (2013).

[4] K. Xiao, Z. S. Guan, G. J. Wang, L. Jiang, D. B. Zhu, and Y. R. Wang, Appl. Phys. Lett. **85**, 1934 (2004).

[5] N. R. Moody, D. P. Adams, M. J. Cordill, D. F. Bahr, and A. A. Volinsky, Symposium on Thin Films, SAND2003-8146C (2003).

[6] A. Lee, C. S. Litteken, R. H. Dauskardt, and W.D. Nix, Acta Materialia **53**, 609 (2005).

[7] M. J. Cordill, N. R. Moody, and D. F. Bahr, Acta Mater. **53**, 2555 (2005).

[8] P. Waters, and A. A. Volinsky, Exper. Mech. **47**, 163 (2007).

[9] A. A. Taylor, M. J. Cordill, L. Bowles, J. Schalko, and G. Dehm, Thin Solid Films **531**, 354 (2013).

[10] P. Goudeau, P. O. Renaul, P. Villain, C. Coupeau, V. Pelosin, B. Boubeker, K. F. Badawi, D. Thiaudière, and M. Gailhanou, Thin Solid Films **398**, 496 (2001).

[11] J. Y. Faou, G. Parry, S. Grachev, and E. Barthel, Phys. Rev. Lett. **108**, 116102 (2012).

[12] H. Y. Yu, C. kim, and S. C. Sanday, Thin Solid Films **196**, 229 (1991).

[13] A. A Volinsky, Mat. Res. Soc. Symp. Proc. **749**, W10.7.1 (2003).

[14] A. A.Volinsky, J. B. Vella, and W. W.Gerberich, Thin Solid Films **429**, 201 (2003).

[15] Z. J. Liu, N. Jiang, Y. G. Shen, and X. Li, Thin Solid Films **516**, 7609 (2008).

[16] J. P. McDonald, V. R. Mistry, K. E. Ray, and S. M. Yalisove, Appl. Phys. Lett. **88**, 183113 (2006).

[17] J. Y Faou, G. Parry, S. Grachev, and E. Barthel, J. Mech. Phys. Solids **75**, 93 (2015).

[18] S. Y. Grache, A. Mehlich, J. D. Kamminga, E. Barthel, and E. Søndergård, Thin Solid Films **518**, 6052 (2010) 4.

[19] G. Gilles, and B. Rau, Thin Solid Films **120**, 109 (1984).

[20] S. B. Iyer, K. S. Harshavardhan, and V. Kumar, Thin Solid Films **256**, 94 (1995).

[21] M. W. Moon, H. M. Jensen, J. W. Hutchinson, K. H. Oh, and A. G. Evans, J. Mech. Phys. Solids **50**, 2355 (2002).

[22] D. He, W. Cheng, J. Qin, J. Yue, E. Xie, and G. Chen, Appl. Surf. Sci. **191**, 338 (2002).

[23] X. D. Zhu, K. Narumi, and H. Naramoto, J. Phys.: Condens. Matter **19**, 236227 (2007).

[24] S. Peponas, M. Lejeune, S. Charvet, M. Guedda, and M. Benlahsen, Surf. Coat. Technol. **212**, 229 (2012).

[25] M. D. Thouless, J. Am. Ceram. Soc. **76**, 2936 (1993).

[26] S. J. Yu, M. G. Chen, J. Chen, H. Zhou, Y. J. Zhang, and P. Z. Si, Surf. Coat. Technol. **228**, 258 (2013).

[27] S. J. Yu, Y. C. Shi, M. G. Chen, P. Z. Si, Y. Zhou, X. F. Zhang, J. Chen, H. Zhou, and Z. W. Jiao, Surf. Coat. Technol. **232**, 884 (2013).

[28] Y. Ni and A.K. Soh, Acta Mater. **69**, 37 (2014).
